# Early Gelatinase Activity Is Not a Determinant of Long-Term Recovery after Traumatic Brain Injury in the Immature Mouse

**DOI:** 10.1371/journal.pone.0143386

**Published:** 2015-11-20

**Authors:** Bridgette D. Semple, Linda J. Noble-Haeusslein, Major Gooyit, Kayleen G. Tercovich, Zhihong Peng, Trung T. Nguyen, Valerie A. Schroeder, Mark A. Suckow, Mayland Chang, Jacob Raber, Alpa Trivedi

**Affiliations:** 1 Department of Neurological Surgery, University of California San Francisco, San Francisco, California, United States of America; 2 Department of Medicine (Royal Melbourne Hospital), Melbourne Brain Centre, University of Melbourne, Parkville, Victoria, Australia; 3 Department of Physical Therapy and Rehabilitation, University of California San Francisco, San Francisco, California, United States of America; 4 Department of Chemistry and Biochemistry, University of Notre Dame, Notre Dame, Indiana, United States of America; 5 Freimann Life Sciences Center and Department of Biological Sciences, University of Notre Dame, Notre Dame, Indiana, United States of America; 6 Departments of Behavioral Neuroscience, Neurology, and Radiation Medicine, Division of Neuroscience, Oregon Health & Science University, Portland, Oregon, United States of America; University of Florida, UNITED STATES

## Abstract

The gelatinases, matrix metalloproteinases (MMP)-2 and MMP-9, are thought to be key mediators of secondary damage in adult animal models of brain injury. Moreover, an acute increase in these proteases in plasma and brain extracellular fluid of adult patients with moderate-to-severe traumatic brain injuries (TBIs) is associated with poorer clinical outcomes and mortality. Nonetheless, their involvement after TBI in the pediatric brain remains understudied. Using a murine model of TBI at postnatal day 21 (p21), approximating a toddler-aged child, we saw upregulation of active and pro-MMP-9 and MMP-2 by gelatin zymography at 48 h post-injury. We therefore investigated the role of gelatinases on long-term structural and behavioral outcomes after injury after acute inhibition with a selective gelatinase inhibitor, *p*-OH SB-3CT. After systemic administration, *p*-OH SB-3CT crossed the blood-brain barrier at therapeutically-relevant concentrations. TBI at p21 induced hyperactivity, deficits in spatial learning and memory, and reduced sociability when mice were assessed at adulthood, alongside pronounced tissue loss in key neuroanatomical regions. Acute and short-term post-injury treatment with *p*-OH SB-3CT did not ameliorate these long-term behavioral, cognitive, or neuropathological deficits as compared to vehicle-treated controls, suggesting that these deficits were independent of MMP-9 and MMP-2 upregulation. These findings emphasize the vulnerability of the immature brain to the consequences of traumatic injuries. However, early upregulation of gelatinases do not appear to be key determinants of long-term recovery after an early-life injury.

## Introduction

Matrix metalloproteinases (MMP)-2 and MMP-9 are members of a family of multi-functional gelatinases. In general, MMPs play an important role in normal brain development, participating in a wide range of physiological processes including embryological modeling, wound healing, angiogenesis, bone remodeling, ovulation and implantation [1–4]. However, these proteases are also key determinants of secondary damage after traumatic and ischemic insults to the adult brain [5–7]. During the acute post-injury phase, MMPs mediate disruption of the blood-brain barrier (BBB), transmigration and infiltration of leukocytes, cerebral edema and oxidative stress [7–13]. In the chronic phase after a brain insult, diverse functions of gelatinases have been proposed, including the modulation of angiogenesis, glial scar formation, myelination and axonal regeneration [5,9,14]. In general, however, an early elevation of gelatinase activity is believed to be primarily detrimental to the injured brain [7]. Clinically, high levels of MMP-2 and MMP-9 are detected acutely post-injury in plasma, brain extracellular fluid and cerebrospinal fluid of adult patients with moderate-to-severe TBI and subarachnoid hemorrhage [15–17], and are associated with poorer outcomes including a longer stay in the intensive care unit and increased risk of mortality [18–20].

In light of the multi-factorial functions of MMPs in the brain, it is important to understand their precise involvement in the injured pediatric brain, including their capacity to influence long-term outcomes. In the adult rodent brain, neuroprotection afforded by the inhibition of MMP-9 activation has previously been demonstrated after focal cerebral ischemia, stroke, and TBI, either by using MMP-9-deficient animals or pharmacological inhibition [8,10,21–30]. In contrast to the adult brain, little is known about the relative contributions of MMPs to secondary pathogenesis and reparative events after early life brain injuries. Similar to the adult brain, models of neonatal hypoxic-ischemic (HI) injuries in p5-p9 rodent pups have yielded findings of upregulated gelatinase expression within 24 h after injury [31–36]. Increased MMP-9 expression after HI in neonate rat pups results in the formation of vasogenic edema and damage to neurons, thereby aggravating secondary brain damage [37]. Further, MMP inhibition early in life with a broad-spectrum inhibitor, GM6001, in p7 rat pups that received HI resulted in amelioration of acute cell death [35], and the preservation of ipsilateral brain tissue loss [32]. Of note, the majority of these studies have focused on only acute and sub-acute time points after injury, and modeling of injury at either neonatal or adult ages. The present study was designed to investigate the role of MMP-9 and MMP-2 in a pediatric (p21) mouse model of TBI, to specifically evaluate the long-term effects of acute, targeted gelatinase inhibition on neurobehavioral outcomes at adulthood.

In humans, injury to the brain during early childhood results in neurocognitive and psychosocial impairments, which may emerge over time and persist long-term [38,39]. We have therefore established a murine model of TBI at p21, approximating a toddler-aged child, which mirrors these unique features, including persistent behavioral sequelae and progressive lesion expansion over development to adulthood [40–42]. Using this model, we have previously demonstrated a reduced antioxidant capacity in the immature injured brain compared to the adult, rendering it particularly vulnerable to the deleterious consequences of elevated gelatinase activity [43,44]. In addition, the infiltration of neutrophils is uniquely exacerbated by TBI to the immature brain, across a prolonged time course compared to injury at adulthood [45]. Infiltrated neutrophils rapidly release active MMP-9 by degranulation in a manner unique from most other cell types, whereby MMP-9 is not complexed with tissue inhibitor of matrix metalloproteinase 1 (TIMP-1), a key endogenous regulator of this potent protease [46,47]. Based upon these data, as well as evidence that the gelatinases play a central role in pathogenesis after TBI to the adult and HI in the neonate brain, we hypothesized that MMPs are likely key determinants of long-term structural and functional recovery after traumatic injury to the pediatric brain.

One promising compound to pharmacologically target gelatinases is SB-3CT, a first-generation, highly-selective, thiirane mechanism-based inhibitor, with *K*_i_ values of 28 ± 7 nM for MMP-2 and 400 ± 150 nM for MMP-9 [48–50]. By targeting only the active forms of MMP-2 and MMP-9, without affecting other MMPs or related proteins, treatment with SB-3CT was neuroprotective after experimental brain injuries at adulthood [23–26]. Following administration, SB-3CT is metabolized to the more potent inhibitor *p*-OH SB-3CT, which possesses *K*_i_ values of 6 ± 3 nM and 160 ± 80 nM for MMP-2 and MMP-9, respectively [49]. We therefore utilized *p*-OH SB-3CT to evaluate the role of MMP-2 and MMP-9 in the injured p21 mouse brain. Based on the early upregulation of MMP-2 and MMP-9, we limited treatment to a period of 48 h post-injury, in order to minimize potential off-target effects on ongoing brain development processes at this time, given the known roles that these gelatinases play in postnatal synaptic maturation [51,52]. Pediatric TBI results in progressive neurodegeneration and emergence of functional deficits over time, hence, we focused our outcome measures on the evaluation of long-term neuroanatomical changes and clinically-relevant behavior outcomes.

## Materials and Methods

### Animals

Male C57Bl/6J pups aged p17 with an accompanying lactating mother were purchased from The Jackson Laboratory (Sacramento, CA) and housed in the Laboratory Animal Resource Center at UCSF Parnassus for 4 days prior to surgery. For pharmacokinetic studies, mice were housed at the Freimann Life Sciences Center at the University of Notre Dame. Standard rodent chow and tap water were available *ad libitum*, and the housing room was maintained on a 12 hour light/dark cycle at approximately 20°C. All procedures involving animals were conducted in accordance with the NIH Guidelines for the Care and Use of Laboratory Animals and approved by the UCSF and University of Notre Dame Institutional Animal Care and Use Committee (IACUC).

### Experimental Design

A total of 112 mice were used for this study, and randomization and blinding were applied to all experiments (S1 Fig). Long-term experiments for behavioral, cognitive, and histological analyses at adulthood (2 months post-injury; ~ 3 months of age at assessment) were conducted across three independent cohorts, with 5 mice per treatment group in each cohort, for a total n = 15 per group (sham-vehicle, sham-drug, TBI-vehicle and TBI-drug).

### Controlled cortical impact model

The controlled cortical impact model of TBI was used as previously described [40,41,53]. TBI to the p21 mouse approximates a toddler-aged child, based upon the structural, biochemical and behavioral characteristics of this age [54]. Pups were anaesthetized with 1.25% 2,2,2-tribromoethanol (Avertin; Sigma-Aldrich, St. Louis, MO) in isotonic saline (i.p. bolus at 0.02 mL/g body weight). After craniotomy, mice were subjected to a controlled cortical impact at 4.5 m/s velocity and 1.73 mm depth of penetration, for a sustained depression of 150 ms, using a 3.0 mm convex impactor tip [40,55]. Mice were maintained on a water-circulating heating pad throughout surgery and recovery. Following impact, the scalp was closed with sutures and each animal administered 0.5 ml of isotonic saline subcutaneously (s.c.) to prevent post-operative dehydration. Sham-operated mice underwent identical surgical procedures, including craniotomy, without receiving the cortical impact. All mice were closely monitored for 1–2 hours post-surgery until return of the righting reflex and full mobilization. Mice were then returned to group-housing, and weighed post-surgery at days 1, 3, and weekly thereafter till the end of the study.

### Treatment with *p*-OH SB-3CT

**Fig 1:**
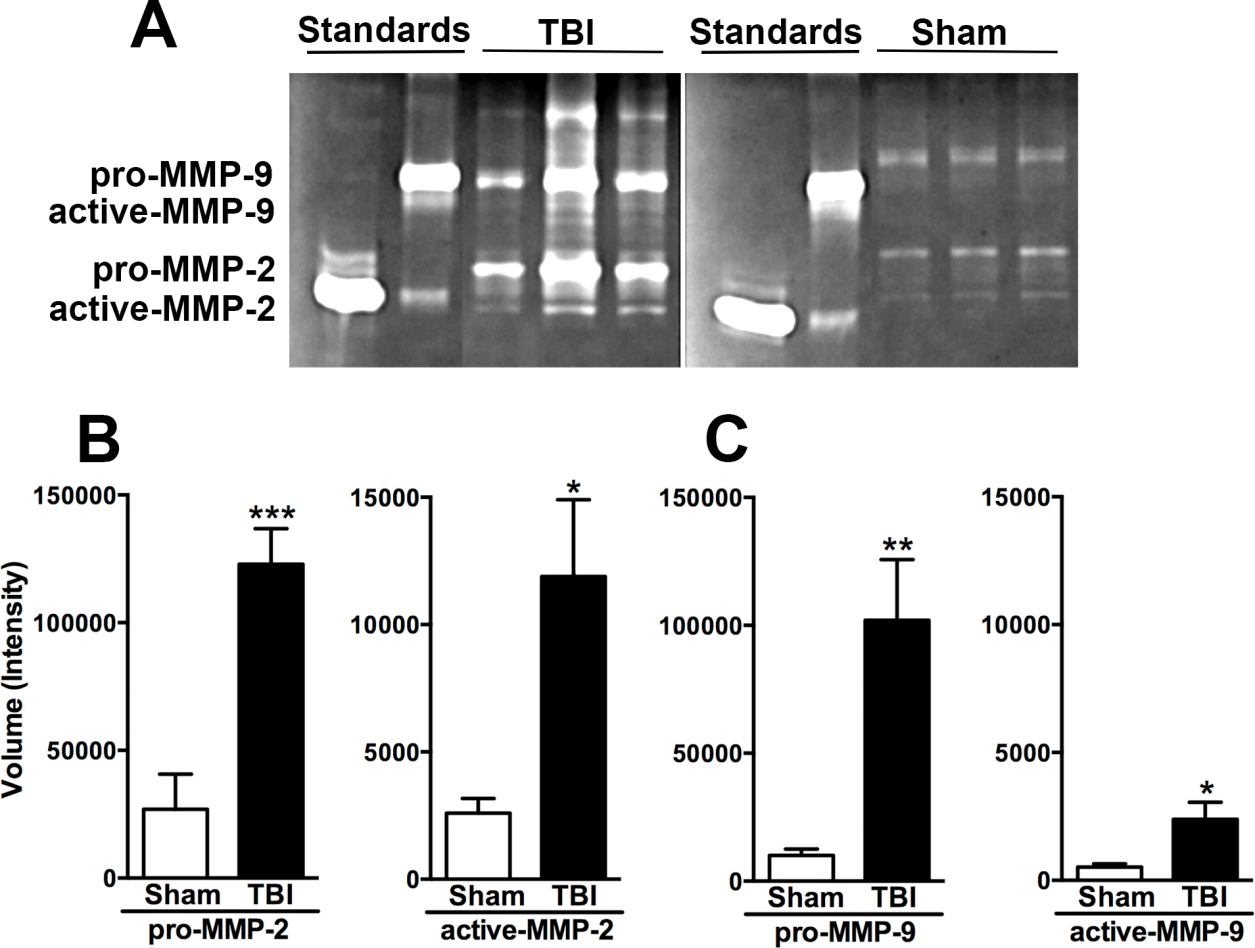
Enhanced gelatinases detected at 48 h after TBI. (A) Gelatin zymography from representative brain lysates indicates increased expression of MMP-9 and MMP-2 at 48 h post-injury as compared to sham controls. The pro-forms of MMP-9 and MMP-2 were detected at ~105 and 72 kDa, respectively. Purified human MMP-2 and MMP-9 were used as standards. (B) Quantification of band intensity revealed a robust increase in the pro-enzyme forms of MMP-2 and (C) MMP-9, as well as increases in the active enzyme forms after TBI compared to sham (n = 6 per group; unpaired t-tests, *p<0.05, **p<0.01 and ***p<0.001, TBI compared to sham). Bars represent mean + sem.

All sham and brain injured animals were treated with 25 mg/kg *p*-OH SB-3CT or vehicle (65% propylene glycol-35% water, s.c) at 2, 4, 24 and 48 h post-injury. The treatment regime was based upon several considerations. Firstly, an equivalent dose of 25 mg/kg of the parent compound SB-3CT was recently shown to be effective at improving functional and neuropathological outcomes after TBI in adult mice [25]. Secondly, an acute time course was chosen in light of previous studies indicating upregulated gelatinases within the first 24 h after injuries in the adult and neonatal brain [25,32,33,35], as well as our own observation of elevated MMP-2 and MMP-9 at 48 h after injury to the p21 brain (see Fig 1). Thirdly, we limited treatment to a period of 48 h post-injury in order to minimize potential off-target effects on ongoing brain development processes at this time, given the known roles that these gelatinases play in postnatal synaptic maturation [51,52]. For acute studies (gelatin zymography and histology) animals were euthanized at 4 hours and for *in situ* zymography, they were euthanized 1 hour after the last injection. For long-term studies (behavioral and cognitive assessments and histology) animals were euthanized at ~2 months post-injury. Euthanasia was by overdose with 2.5% Avertin i.p. followed by decapitation or cardiac transperfusion as described below.

### Gelatin zymography

Flash-frozen brain samples (n = 6/group) were weighed and washed three times with phosphate buffered saline (PBS) to remove external contaminants. Samples were homogenized with ten volumes of cold lysis buffer (25 mM Tris-HCl pH 7.5, 100 mM NaCl, 1% v/v Nonidet P-40 and complete protease inhibitors, without EDTA) using a Bullet Blender (Next Advance, Inc., Averill Park, NY) for 5 min. The homogenates were centrifuged at 15000×*g* for 10 min at 4°C, and 150 μL aliquots of the supernatants were concentrated by affinity precipitation with gelatin-agarose beads. The bound gelatinases were released from the beads in 2% SDS and samples were analyzed by electrophoresis in a 10% gelatin zymogram gel, as previously described [56]. After electrophoresis, gels were incubated at room temperature for 30 min in renaturing solution (2.5% v/v Triton X-100) with gentle agitation, washed with water to remove Triton X-100, incubated at room temperature for 30 min in developing buffer (50 mM Tris–HCl, 5 mM CaCl_2_, 0.2 M NaCl, and 0.02% v/v Brij 35, pH 7.8) with gentle agitation, transferred to fresh developing buffer, then incubated at 37°C for an additional 40 h. Areas of proteolytic activity were visualized as clear bands against a blue background after 1 h staining with Coomassie brilliant blue. Densitometric analysis to quantify the relative activity of the gelatinases was performed with Gel Doc^TM^ EZ Imager (Bio-Rad Laboratories, Inc., Hercules, CA).

### Long-term behavioral and cognitive assessments

Mice were randomized for behavior testing and all behavior testing was performed by individuals blinded to injury and treatment. Behavioral and cognitive testing of brain injured mice and sham controls commenced at 2 months post-injury (~ 3 months of age; n = 15/group). An extensive battery of behavioral assessments was performed in the following order: open field, rotarod, elevated plus maze, three-chamber social task, resident-intruder, and Morris water maze (MWM). Mice were placed in individual clean cages 24 h prior to commencement of testing for overnight isolation and habituation. Behavioral equipment was cleaned between mice with 10% bleach followed by 3% acetic acid to eliminate contaminating odors, and testing was conducted between 9 am and 6 pm daily.

#### Open field test

Exploratory behaviors were assessed over a 10 min session in an automated open field arena (40.6 cm x 40.6 cm; Kinder Scientific, Poway, CA). Interfaced Motor Monitor software allowed for calculation of parameters including total distance travelled, and relative time spent in the center versus the periphery [41,53].

#### Rotarod

The accelerating rotarod (Ugo Basile 7650, Comerio VA) was performed to assess general motor function, coordination and motor learning, as previously described [41,53]. The latency to fall was recorded in sec, and mice were tested across three consecutive days, three trials per day with an inter-trial interval of approximately 30 min.

#### Elevated plus maze

The elevated plus maze (Kinder Scientific, Poway, CA) assesses anxiety based upon the natural tendency of rodents to avoid the open arms in preference for enclosed areas [57]. Mice were placed individually on the apparatus and allowed free access for a 10-min period. Times spent in the open versus closed arms was assessed as previously described [41,53].

#### Resident-intruder test

To assess social investigative behaviors, a novel stimulus mouse (matched for strain, age and sex) was presented into the established home cage of the test mouse, and investigative behaviors were quantified from video recordings as previously described [42,58].

#### Three-chamber social approach task

The three-chamber paradigm allows for the evaluation of social affiliation and social recognition in mice [59–61]. The task was performed as previously described [42]. In brief, three consecutive stages of 10 min each allow for (1) habituation in an empty 3-chambered arena; (2) a choice between an empty cup and a cup containing a novel stimulus mouse; and (3) a choice between a second novel stimulus mouse and the first, now familiar mouse. Stages 2 and 3 are measures of the test mouse’s preference for sociability and social recognition, respectively. ‘Stimulus’ mice used for the three-chamber task were age-matched naïve male C57Bl/6J mice. Data were expressed as time spent in each chamber (% of total time).

#### Morris water maze (MWM)

The MWM was used to assess spatial learning and memory as previously described [41,53,62], using a 140 cm-diameter circular pool filled with opaque water (22°C). Mice underwent two daily sessions (spaced 3.5 h apart) for 5 consecutive days. Each session consisted of three 60-sec trials with a 10–15 min inter-trial interval. During days 1 and 2, the platform was raised above the water surface and clearly labeled with a flag (‘visible platform’). The platform location was rotated to a different quadrant during this training stage. Mice that failed to reach the platform within 60 sec were guided there by the investigator. During days 3, 4 and 5 (‘hidden platform’), the platform was hidden below the water surface and maintained in a constant location, such that mice were required to use spatial cues from the room to locate it. Movements were tracked with an overhead mounted video camera interfaced with Noldus EthoVision software (Noldus IT, Tacoma, WA), for quantification of the cumulative distance to and latency to locate the platform, as well as swim velocity (cm/s). At the conclusion of days 3, 4 and 5, ~ 1 h after completion of the last trial for that day, the platform was removed from the pool and a 60 sec ‘probe trial’ was conducted for each mouse. A fourth probe trial was performed one week later. As mice typically showed a preference for the target quadrant early in the probe trials and subsequently searched elsewhere, we analyzed only the first 30 seconds of each probe trial using cumulative distance to the target as an outcome measure.

### Stereological assessments at adulthood

Upon completion of behavioral and cognitive assessments at ~ 2 months post-injury, anesthetized mice were perfused transcardially with ice-cold saline followed by 4% paraformaldehyde in 0.1M PBS. Collected brains were post-fixed overnight in 4% PFA and then transferred into a 30% sucrose solution for 72 h before being embedded in Neg50^TM^ (Richard-Allan Scientific, Thermo Fisher Scientific, Fremont, CA). Serial coronal sections spanning the entire cortex were cut at 20 μm or 40 μm (for time points of 48 h or 2 months post-injury, respectively).

#### Cortical and hippocampal volume measurements

An estimation of intact cortical and hippocampal volumes was performed on 40 μm coronal sections stained with cresyl violet, collected at ~ 2 months post-injury. The unbiased Cavalieri method was performed with StereoInvestigator software (MicroBrightField v10.21.1, Williston, VT, USA), using a Nikon E600W microscope configured with a motorized stage, MAC 5000 controller and Retiga 2000R color digital camera (QImaging, Surrey, BC, Canada). Spanning Bregma 1.5 to -3.8 mm, 12–16 sections containing cortex and 7–10 sections containing hippocampus were assessed per brain, using a sampling interval of 8 and a grid size of either 200 μm or 75 μm for cortex and hippocampus, respectively. Measurements were confined to the dorsal hemispheres as previously described [53,58] using a 2x objective for the cortex and 4x for the hippocampus. The Gundersen mean coefficient of error (m = 1) was maintained ≤0.03 for cortical volume estimates and ≤0.08 for the hippocampus and dentate gyrus (DG). Group means are expressed as estimated volume (mm³). Certain sections were excluded from stereological analyses due to poor tissue integrity, folding or incomplete staining. In these cases, the section was deemed to be ‘missing’ and estimation for this section was calculated from the StereoInvestigator program based upon the adjacent sections. This was permitted for a maximum of 2 non-adjacent sections per brain. Final group sizes for this analysis were therefore a subset of those used for behavioral assessment at this chronic time point (n = 11–13 for cortex; n = 13–15 for hippocampus and dentate gyrus).

#### Hippocampal cell counts

The optical fractionator method was used to obtain an unbiased estimate of the total number of hippocampal neurons in the injured (ipsilateral) DG using StereoInvestigator software [63,64]. Prior to cell counts, sections were evaluated for quality as described above for volume measurements, resulting in final numbers of n = 8–9 per group. Using a sampling interval of 4, approximately 10 sections per brain were stained with cresyl violet and key regions of interest (ipsilateral upper and lower DG blades) were contoured using a 4x objective. Cell counts were performed using a 60x oil immersion objective, with a dissector counting frame of 20 x 20 μm and a grid size of 80 x 80 μm. The actual mounted thickness was determined at every counting site, and a dissector height of 10 μm with a top guard zone of 1 μm allowed for quantification of cells in a three-dimensional manner. The Gundersen mean coefficient of error (m = 1) for individual estimates was maintained at <0.10. The total number of cells per contoured region was estimated by the following equation: N = ∑Q · (t/h)(1/asf)(1/ssf); where Q is the number of cells counted, t is the measured section thickness, h is the dissector height, asf is the area sampling fraction, and ssf is the section sampling fraction [65].

### Pharmacokinetics of *p*-OH SB-3CT

*p*-OH SB-3CT was synthesized as described previously [49] and formulated in 65% propylene glycol/35% water at a concentration of 5.0 mg/mL. For the pharmacokinetic (PK) study, *p*-OH SB-3CT was administered s.c. to p21 naïve male C57Bl/6J mice at 25 mg/kg at 0, 2 and 24 h, and plasma and brains were collected at 0.5, 1, 2 and 4 h after the final dose. Dosing route and concentration were similar to what we proposed to do for treatment for the current study. Terminal blood samples were collected in heparinized tubes through the posterior vena cava and centrifuged to obtain plasma (n = 3 per time point). Whole brain samples were harvested after transcardial perfusion with saline, immediately flash frozen in liquid nitrogen, and stored at -80°C until analysis. Sample processing and quantification of *p*-OH SB-3CT in brain and plasma for PK analyses were conducted as previously reported [66].

### *In situ* zymography

Mice received either sham or injury and were treated with vehicle or *p*-OH SB-3CT (n = 4/group), at the same dosage and timing as for all other studies (S1 Fig). One hour after the last injection, brains were collected and embedded in optimal cutting temperature medium (OCT, Tissue-Tek, CA). The tissue blocks were cryosectioned at 10μm thickness and subjected to *in-situ* zymography, as described by Oh *et al*. [30]. Briefly, the sectioned tissues were stained with DQ-gelatin (Molecular Probes, Inc., OR, USA) to detect gelatinase activity, followed by incubation with DAPI solution to detect nuclei. The images for *in-situ* zymography were obtained using same exposure setting for all sections examined by confocal microscopy (Nikon Eclipse 90*i* Fluorescent Microscope (Nikon Instruments Inc., Melville, NY). No image processing was performed to adjust signal intensities of DQ-gelatin.

### Acute histological assessments

Brain injured animals and sham controls were euthanized at 2 d post-injury. Dying cells were detected by double-labeling for terminal deoxynucleotidyl transferase-mediated dUTP nick 3’-end labeling (TUNEL) in combination with immunofluorescence for activated (cleaved) caspase-3, a hallmark of apoptosis. The *in situ* Cell Death Detection kit was performed according to the manufacturer’s instructions (Roche Diagnostics, Indianapolis, IN). Non-specific binding was blocked by application of a 10% normal goat serum solution containing 1% bovine serum albumin in PBS for 1 h, followed by overnight incubation with a rabbit polyclonal anti-activated caspase-3 antibody (1:500; Millipore, Billerica, MA). Positive immuno-labeling was detected by subsequent application of a Cy-3 conjugated goat anti-rabbit IgG antibody (1:1600; Jackson Immunoresearch, West Grove, PA), and nuclei were counterstained by application of ProLong® Gold antifade mounting media (Thermo Fisher Scientific, Pittsburgh, PA) containing 4',6-diamidino-2-phenylindole (DAPI). Images of the stained sections were captured using a Nikon Eclipse 80*i* fluorescent microscope and SPOT^TM^ Imaging Solutions software (Diagnostics Instruments, Sterling Heights, MI). Eight sections per brain (120 μm interval) were examined, starting with the anterior emergence of hippocampus and spanning the entire lesion (n = 6/group). Non-overlapping images were captured at 20x objective of the ipsilateral dorsal cortex and hippocampus, using the ventral edge of the third ventricle to create a horizontal dorsal boundary. Immuno-positive cells with DAPI-positive nuclei were counted using Metamorph analysis software (Molecular Devices, Sunnyvale, CA).

### Statistical analyses

Statistical analyses were performed using Prism v.6.0 (GraphPad Software, Inc., La Jolla, CA) and SPSS software (Chicago, IL), with a significance level of p<0.05. For comparison between two key groups of interest (e.g. TBI vehicle vs. TBI drug-treated), unpaired t-tests were used to assess gelatinase activity, volumetric tissue loss, and cell death. For data that were not normally distributed, a Mann-Whitney test was performed. Two-way analysis of variance (ANOVA) was used to compare two or more groups or factors (injury, drug treatment and/or time). When the interaction between factors was not statistically significant, main effects are only reported. *A priori* planned comparisons evaluated potential differences between key groups of interest (e.g. TBI vehicle vs. TBI drug-treated), or *post-hoc* analyses where appropriate. Repeated measures (RM) were used for the rotarod and three-chamber tasks, with a between-subjects factor of injury and a within-subjects factor of session or chamber. MWM data were analyzed using multivariate or RM ANOVA with injury and treatment as between subject factors. Results are expressed as mean + or ± standard error of the mean (sem).

## Results

### MMPs are upregulated in the brain after injury at p21

We first asked if MMPs were upregulated acutely after injury to the immature brain. Lysates of the ipsilateral cortex, collected at 48 h after TBI, were analyzed by gelatin zymography (Fig 1A). Compared to sham controls, levels of pro-MMP-2 and pro-MMP-9 were significantly elevated after TBI, by ~4.6 fold (unpaired t-test; p = 0.0006) and ~10 fold (p = 0.0032), respectively (Fig 1B and 1C). The active enzyme forms were also increased in the injured brain compared to shams by ~4.6 fold for both MMP-2 (p = 0.0128) and MMP-9 (p = 0.0200). These findings provide evidence that both pro and active forms of MMP-2 and MMP-9 were elevated in the p21 mouse brain at 48 h post-injury.

### Acute gelatinase inhibition does not alter long-term, injury-induced hyperactivity, cognitive and social deficits

After surgery at p21, body weights were monitored as an indicator of overall health across development (S2A Fig). All mice gained weight over time (2-way RM ANOVA, effect of time; p<0.0001), regardless of injury or drug treatment (effect of group; p>0.05), indicating that mice maintained good general health throughout the study.

**Fig 2:**
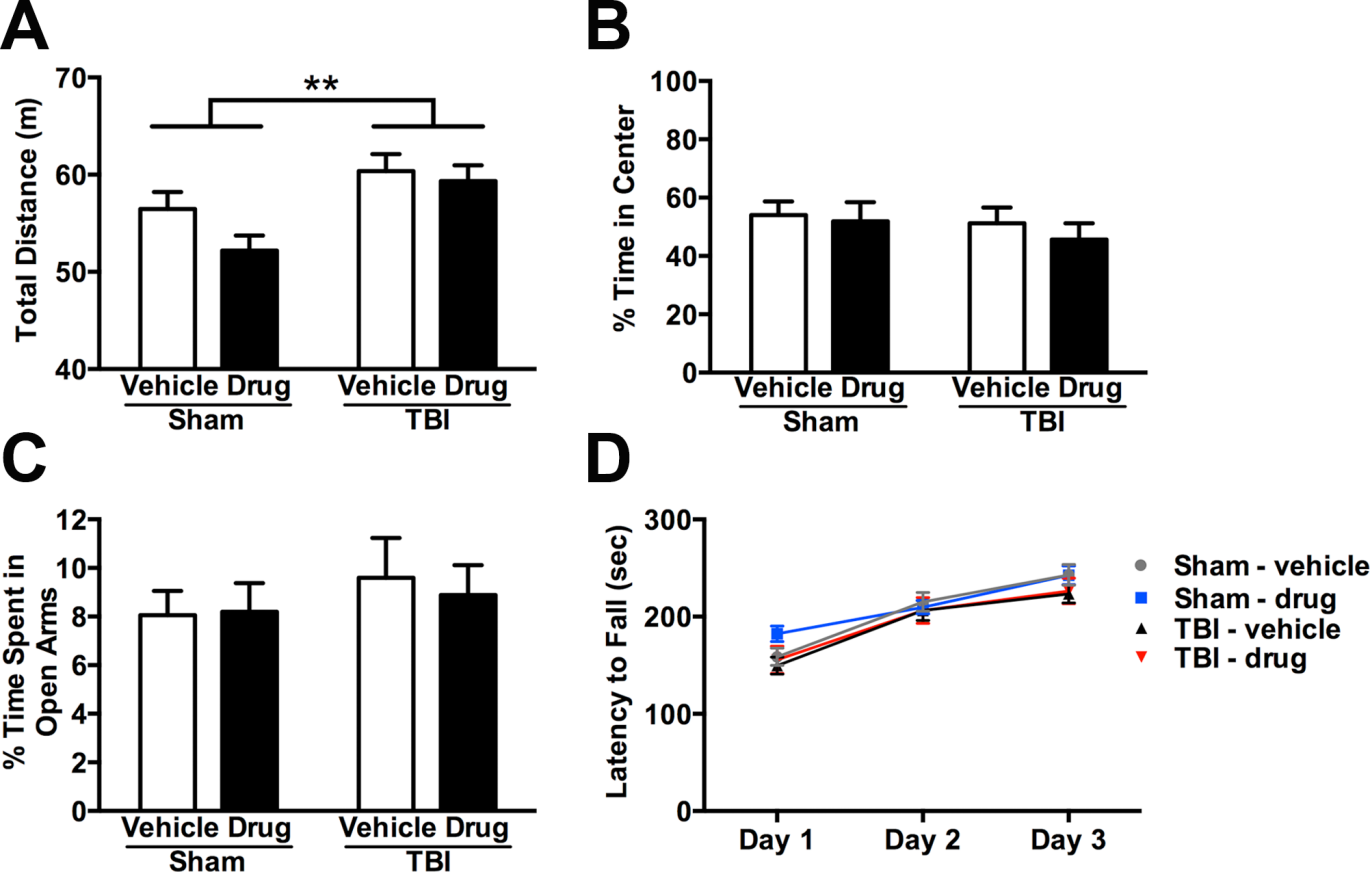
Early gelatinase inhibition does not impact injury-induced hyperactivity, measures of anxiety, or motor function at adulthood after pediatric TBI. (A) Injury resulted in hyperactivity by adulthood, indicated by increased total distance traveled in an open field (2-way ANOVA, effect of injury, **p<0.01). (B) The percent time spent in center of the open field, a measure of anxiety, was not altered by either injury or treatment (2-way ANOVA n.s.). (C) Anxiety was also measured by percent time spent in the open arms of the elevated plus maze, and neither injury nor *p*-OH SB-3CT treatment affected this measure (2-way ANOVA n.s.). (D) Motor function, evaluated by latency to fall from an accelerating rotarod, was similar in all groups across consecutive testing days (2-way RM ANOVA). Bars represent mean + sem and values represent mean ± sem (n = 15/group).

A battery of behavioral and cognitive assessments were conducted at 3 months of age (~ 2 months post-injury), to determine whether the acute inhibition of gelatinases modulated long-term behavioral outcomes when mice had reached adulthood. The open field test was employed to evaluate general activity and anxiety. Consistent with previous studies [41,53], TBI at p21 resulted in hyperactivity by adulthood, as detected by an overall effect of injury by 2-way ANOVA (p = 0.0017; Fig 2A). Treatment with *p*-OH SB-3CT did not ameliorate this hyperactivity (2-way ANOVA overall effect of drug; p = 0.1189). Anxiety-like behavior was evaluated as percentage time spent in the center of the open field (Fig 2B), however, neither TBI nor *p*-OH SB-3CT treatment affected this measure at adulthood (2-way ANOVAs, effect of injury or drug; p>0.1). The elevated plus maze was also used to assess anxiety-like behavior (Fig 2C). In this test, time spent in the exposed open arms was not affected by either TBI or *p*-OH SB-3CT treatment (2-way ANOVAs, effect of injury or drug; p>0.3), in agreement with a lack of anxiety-related behavior observed in the open field arena. In summary, TBI at p21 induced chronic hyperactivity but did not impact anxiety and the inhibition of acute gelatinase activity by *p*-OH SB-3CT treatment had no influence on long-term injury-induced hyperactivity.

Potential motor dysfunction was next evaluated by performance on the accelerating rotarod (Fig 2D). There were no overall differences in latency to fall from the rotarod between the treatment or injury groups across three consecutive days of testing (2-way RM ANOVA, effect of group; p = 0.4468). However, all mice showed significant improvement over time (effect of time; p<0.0001). Of note, although improvements (increased latency to fall) were observed in all groups between day 1 and day 2, only sham-operated mice continued to improve between day 2 and day 3 (Sidak's post-hoc, p<0.05). In contrast, performance by brain-injured mice appeared to plateau by day 2, suggestive of a potential modest deficit in motor learning after TBI.

Task learning and spatial memory were assessed in the MWM at adulthood. Swim velocity during the visible platform trials was not influenced by TBI (multivariate ANOVA, overall effect of injury; p = 0.885) or *p*-OH SB-3CT treatment (effect of drug; p = 0.833; S2B Fig). These data indicate that all mice had similar motor abilities and motivation, and that both latency to reach the platform and cumulative distance to the target can be used as performance measures for group comparisons.

**Fig 3:**
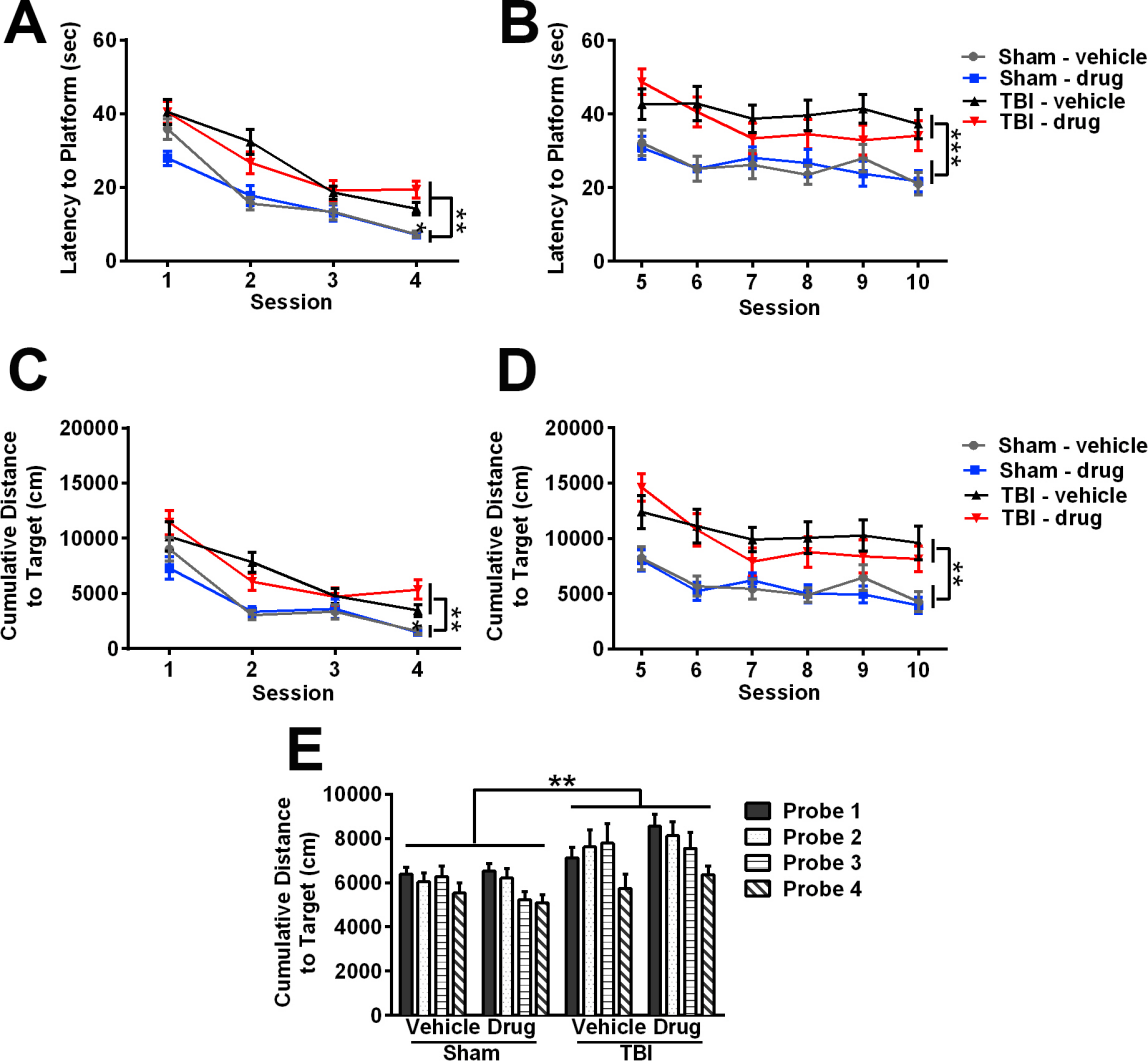
Cognitive deficits detected in the Morris water maze (MWM) at adulthood after pediatric TBI, are unaffected by gelatinase inhibition. (A) During the visible sessions, quantification of latency to reach the platform revealed an impairment in task learning by TBI mice compared to sham controls (multivariate ANOVA overall effect of TBI, **p<0.01). (B) During hidden platform sessions, injured mice also showed a greater latency to reach the platform as compared to sham controls (overall effect of TBI, ***p<0.001), indicating an impairment in spatial memory. Cumulative distance to the target was also quantified as an alternative outcome measure (C-D), which similarly detected impairments in task performance and spatial memory in TBI mice compared to sham controls (overall effect of TBI, **p<0.01). (E) Probe trial performance was quantified as cumulative distance to the target. Injured mice traveled a greater distance to reach the target quadrant compared to sham controls (RM ANOVA, overall effect of TBI, **p<0.01) (n = 15/group). Bars represent mean + sem and values represent mean ± sem.

Next, the learning curves across repeated trials were analyzed using multivariate ANOVA. During the visible platform sessions, TBI mice required more time to reach the platform location, indicating impairments in task learning (multivariate ANOVA overall effect of injury; p<0.01; Fig 3A). For the hidden platform sessions (Fig 3B), TBI also increased latency to reach the platform (multivariate ANOVA effect of injury; p<0.001), indicating deficiency in spatial memory. Treatment with *p*-OH SB-3CT did not modify this injury-related behavior during either the visible (multivariate ANOVA effect of drug treatment; p = 0.529) or hidden platform sessions (multivariate ANOVA effect of drug treatment; p = 0.542). Similar results were obtained when data were analyzed using cumulative distance to the target as an alternative performance measure (Fig 3C and 3D). In agreement with the latency measures, injury significantly increased the distance traveled to reach the platform during both the visible and hidden sessions (multivariate ANOVA, overall effect of injury; p<0.01 for visible and hidden sessions, respectively), with no effect of *p*-OH SB-3CT treatment (overall effect of drug; p>0.4). These data confirm that pediatric TBI impairs the acquisition of task learning and spatial memory at adulthood. However, acute inhibition of gelatinases by *p*-OH SB-3CT after injury did not ameliorate these deficits.

To evaluate spatial memory retention, probe trials were conducted at the end of each of the three hidden platform days, as well as an additional trial completed one week later (for a total of four probe trials), and cumulative distance to the target was quantified for each of the probe trials (Fig 3E). A significant overall effect of injury detected by 2-way RM ANOVA (p = 0.005), in the absence of a drug effect (p = 0.9366), confirmed that the effect of injury on spatial memory retention was not rescued by acute inhibition of gelatinases with *p*-OH SB-3CT.

**Fig 4:**
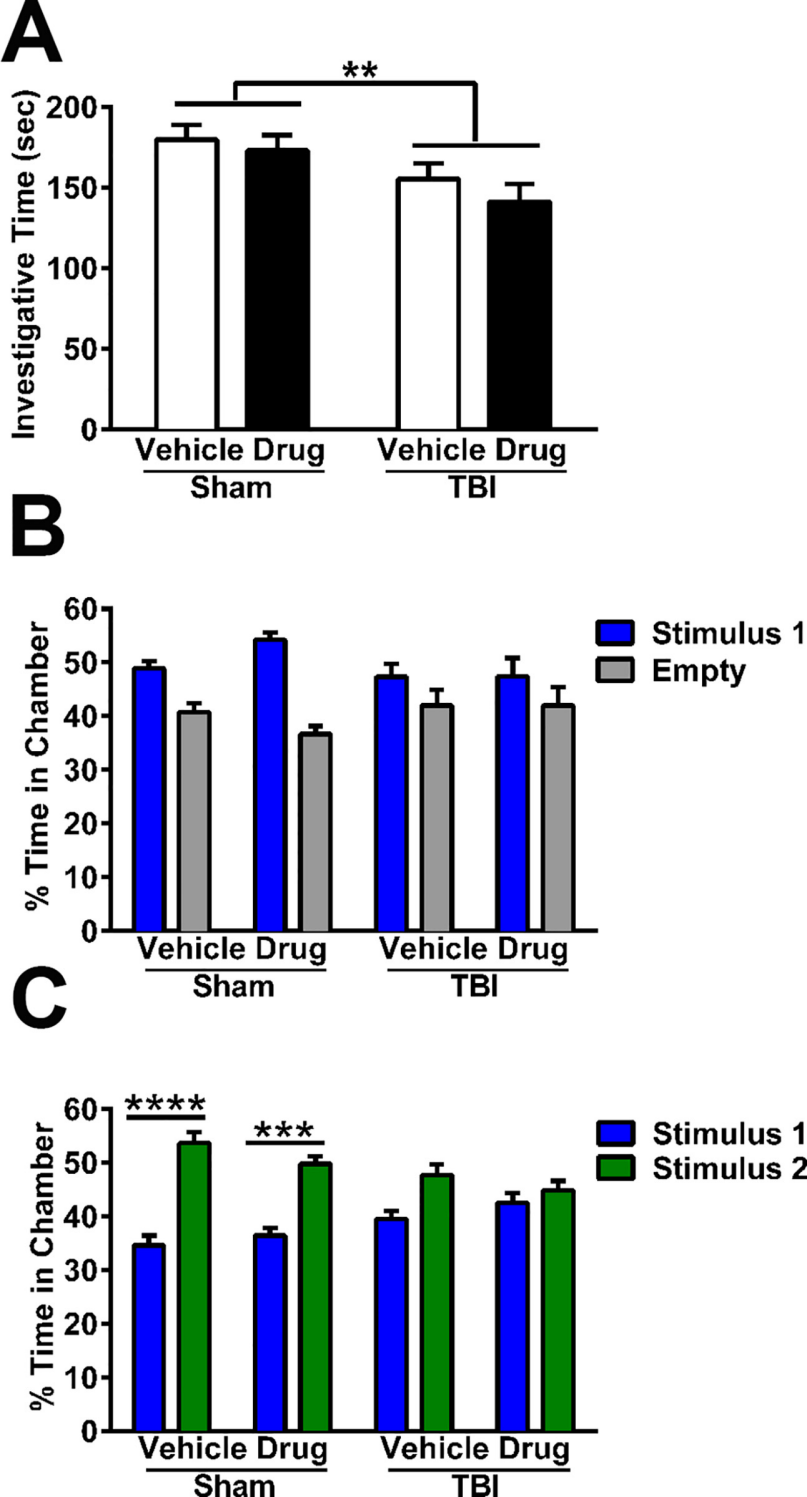
Acute gelatinase inhibitor does not attenuate deficits in social behavior at adulthood after pediatric TBI. (A) Social investigation was quantified by the resident-intruder paradigm, revealing that TBI mice as compared to sham controls spent less time investigating a naïve intruder mouse (2-way ANOVA overall effect of TBI, **p<0.01). (B) In the three-chamber social approach task (stage 2), all mice showed an overall preference for sociability with stimulus mouse 1 compared to the empty chamber (2-way RM ANOVA overall effect of chamber, p = 0.0003). (C) Stage 3 of the three-chamber task tested social novelty. Here, sham-operated mice revealed a preference for a novel stimulus mouse compared to the now-familiar mouse (2-way RM ANOVA interaction, p = 0.0055; subsequent Sidak’s post-hoc tests, ***p<0.001, ****p<0.0001 as indicated graphically). In contrast, TBI mice showed a lack of of social memory (n.s. by Sidak's post-hoc) (n = 15/group). Bars represent mean + sem.

We have previously demonstrated the emergence of social behavior deficits by adulthood after pediatric TBI in this mouse model [42], which parallels commonly observed social dysfunction in young brain-injured patients [67]. We therefore evaluated social behavior by two complementary tests, the resident-intruder task and the three-chamber social approach. In the resident-intruder task, brain-injured mice spent significantly less time than sham-operated controls engaged in social investigation of the intruder mouse (2-way ANOVA overall effect of TBI; p = 0.0063; Fig 4A). This injury-induced reduction in social investigation was independent of treatment with *p*-OH SB-3CT (overall effect of drug; p = 0.2931).

The three-chamber social approach task examines whether mice show a preference for social interaction and/or a preference for social novelty. During stage 1 (habituation to the empty chambers), no interaction or effect of group was observed (2-way RM ANOVA, p>0.9). Unexpectedly, there was an overall preference for spending time in the right-side chamber compared to the left (overall effect of chamber, p = 0.0256), although planned between-group comparisons did not identify specific group differences (p>0.05). To prevent this general, innate side preference from potentially confounding the subsequent stages, positioning of the cups that were either empty or containing stimulus mice were randomly alternated between mice across all groups for subsequent test stages.

Stage 2 provided mice with the choice between chambers containing either an empty cup or a novel stimulus mouse (Fig 4B). Here, all mice showed an overall preference for the chamber containing the stimulus mouse compared to the empty cup (2-way RM ANOVA overall effect of chamber, p = 0.0003), which appeared most evident in sham mice, which typically spent ~50% time with the stimulus mouse compared to ~40% time in the empty chamber. Although both vehicle and *p*-OH SB-3CT-treated TBI mice appeared to spend a more equivalent time in each chamber, no overall differences between groups (injury or drug treatment) were detected by 2-way RM ANOVA (p = 0.5093).

In stage 3, the test mouse was offered a choice between chambers containing the now familiar stimulus mouse (‘stimulus 1’), or a second (novel) mouse (‘stimulus 2;’ Fig 4C). Here we found a significant interaction between chamber and group factors by 2-way RM ANOVA (p = 0.0055). Subsequent within-group, post-hoc analysis revealed that both groups of sham-operated mice spent considerably more time with stimulus mouse 2 compared to stimulus mouse 1 (vehicle-sham: p<0.0001, and *p*-OH SB-3CT-sham: p<0.001), indicating a preference for social novelty. Consistent with previous evidence of a social novelty deficit after pediatric TBI [42], brain-injured mice failed to show a preference for the novel stimulus mouse, (2-way RM ANOVA vehicle-TBI, p>0.05) and this was not rescued by *p*-OH SB-3CT (2-way RM ANOVA *p*-OH SB-3CT-TBI, p>0.05). In summary, acute gelatinase inhibition did not rescue TBI-mediated chronic social dysfunction at adulthood.

### Prominent loss of cortical and hippocampal tissue is not rescued by gelatinase inhibition

Upon completion of the behavioral assays, histological sections were collected to determine the effect of acute *p*-OH SB-3CT treatment on injury-induced tissue loss at adulthood. Here, injury resulted in ~ 40% volumetric tissue loss in the cortex and 60% loss in the hippocampus of injured brains compared to shams, encompassing portions of the somatosensory, motor and visual cortices, and dorsal hippocampus.

**Fig 5:**
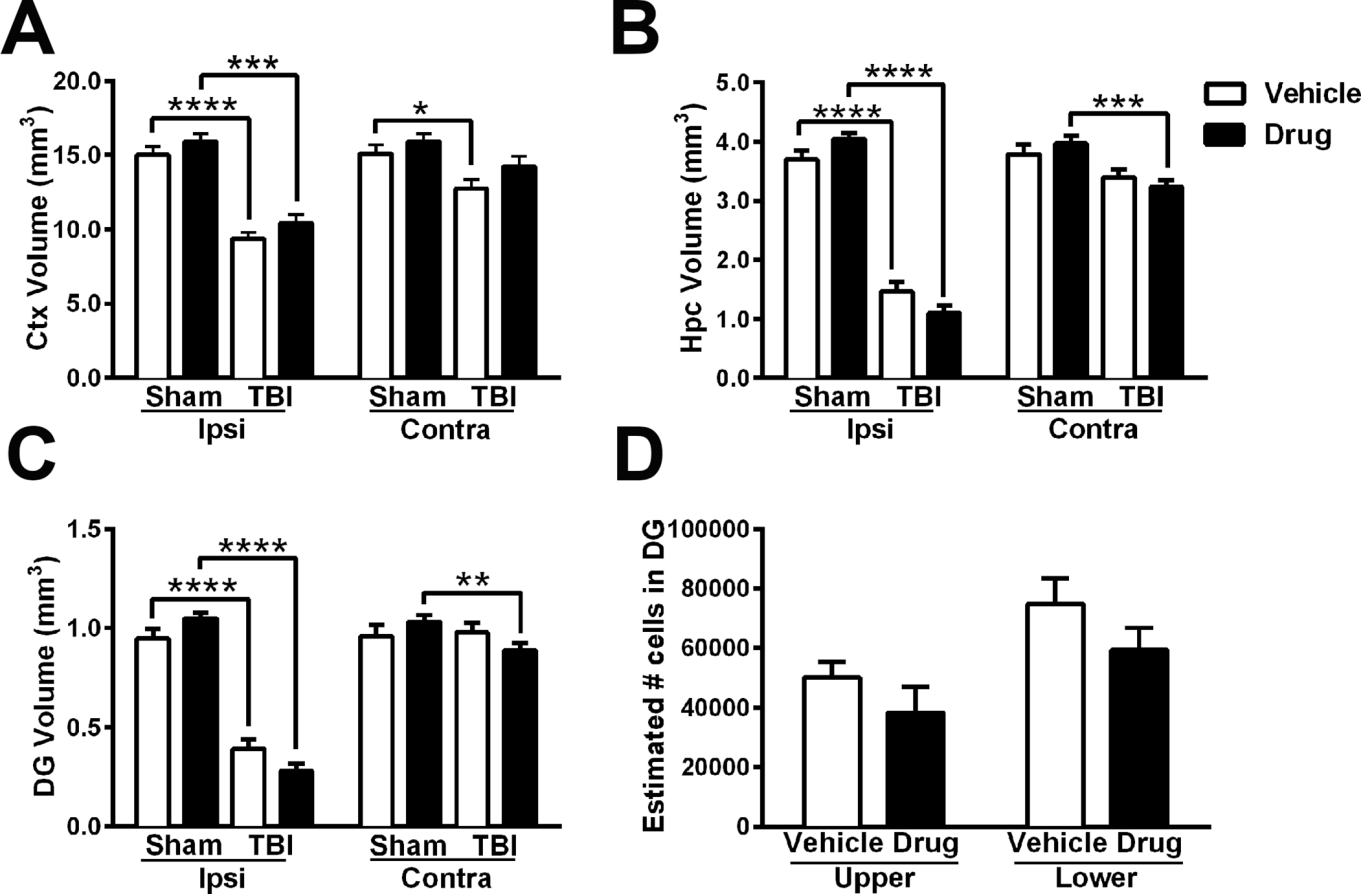
Gelatinase inhibition with *p*-OH SB-3CT does not attenuate extensive injury-induced loss of cortical and hippocampal structures. Volumetric estimates spanning Bregma 1.5 to -3.8mm in the cortex (Ctx; A), hippocampus (Hpc; B) and dentate gyrus (DG; C) revealed injury-induced reductions (unpaired t-tests *p<0.05, **p<0.01, ***p<0.001, and ****p<0.0001 as indicated graphically; n = 11–15/group). (D) Unbiased cell counts performed in the ipsilateral DG found similar numbers of surviving neurons in the upper and lower blades of injured mice independent of drug treatment (n = 8–9/group). Bars represent mean + sem.

In the ipsilateral cortex (Fig 5A), injury caused significant volumetric tissue loss in both vehicle and drug-treated TBI groups compared to their sham controls (unpaired t-tests, p<0.0001 and p<0.001, respectively). Further, drug treatment did not influence ipsilateral cortical volumes in either sham (p = 0.2856) or injured animals (p = 0.1669). In the contralateral cortex, volumetric tissue loss was evident after TBI in vehicle-treated brains as compared to their sham controls (p = 0.0148); however, while cortex volumes in *p*-OH SB-3CT-treated TBI mice appeared to be reduced compared to their sham controls, this did not reach statistical significance (p = 0.0752). When directly comparing TBI groups or sham groups to each other, treatment with *p*-OH SB-3CT did not alter contralateral cortical volumes (unpaired t-tests, p>0.1).

In the ipsilateral hippocampus (Fig 5B), there was a significant volumetric loss in response to injury (TBI vs. sham) in both vehicle-treated (unpaired t-test, p<0.0001) and drug-treated mice (p<0.0001). Sham mice treated with *p*-OH SB-3CT tended to exhibit *larger* hippocampal volumes as compared to vehicle-treated sham mice (p = 0.0669). In contrast, *p*-OH SB-3CT-treated TBI mice displayed *reduced* hippocampal volumes, that did not reach statistical significance as compared to vehicle-treated mice (p = 0.0835). Injury also appeared to impact the contralateral hippocampus, with reduced volumes observed in *p*-OH SB-3CT-treated TBI brains compared to their sham controls (p = 0.0009), although this was not apparent in vehicle-treated mice (p = 0.1755). Direct comparison between vehicle- and drug-treated groups found that *p*-OH SB-3CT did not affect the contralateral hippocampus volume in either sham (p = 0.2844) or TBI brains (p = 0.6792).

Volumetric changes in the DG (Fig 5C) mirrored findings observed in the dorsal hippocampus overall, with a pronounced loss of tissue ipsilateral to the injury (vehicle-treated: p<0.0001; drug-treated: p<0.0001; unpaired t-tests), and no effect of drug treatment in either sham or TBI groups (sham: p = 0.0686; TBI: p = 0.0684). In the contralateral DG, injury-dependent tissue loss was evident in drug-treated mice (p = 0.0079), but not in vehicle-treated mice (p = 0.7829). In both sham and TBI mice, drug treatment did not affect contralateral DG volume (sham: p = 0.2555; TBI: p = 0.1961). Overall, these results indicated a consistent loss of tissue after TBI, most prominent on the side ipsilateral to the impact site. Acute treatment with *p*-OH SB-3CT did not alleviate ipsilateral volumetric tissue loss in either the cortex or hippocampal regions. However, we did observe a modest, non-significant differential effect of *p*-OH SB-3CT treatment on hippocampal volumes in both injured and sham mice.

Lastly, we used the Optical Fractionator method to specifically quantify the number of surviving neurons in the hippocampal DG after TBI (Fig 5D). Here, the granule cell layer of both vehicle and *p*-OH SB-3CT-treated mice contained a similar number of neurons, in both the upper blade (unpaired t-test, p = 0.2752) and lower blade of the DG (p = 0.1888). In summary, acute treatment with *p*-OH SB-3CT did not alter the numbers of surviving DG neurons by adulthood, in alignment with the lack of a significant treatment effect observed by volumetric analysis.

### Pharmacokinetics and brain distribution of *p*-OH SB-3CT

**Fig 6:**
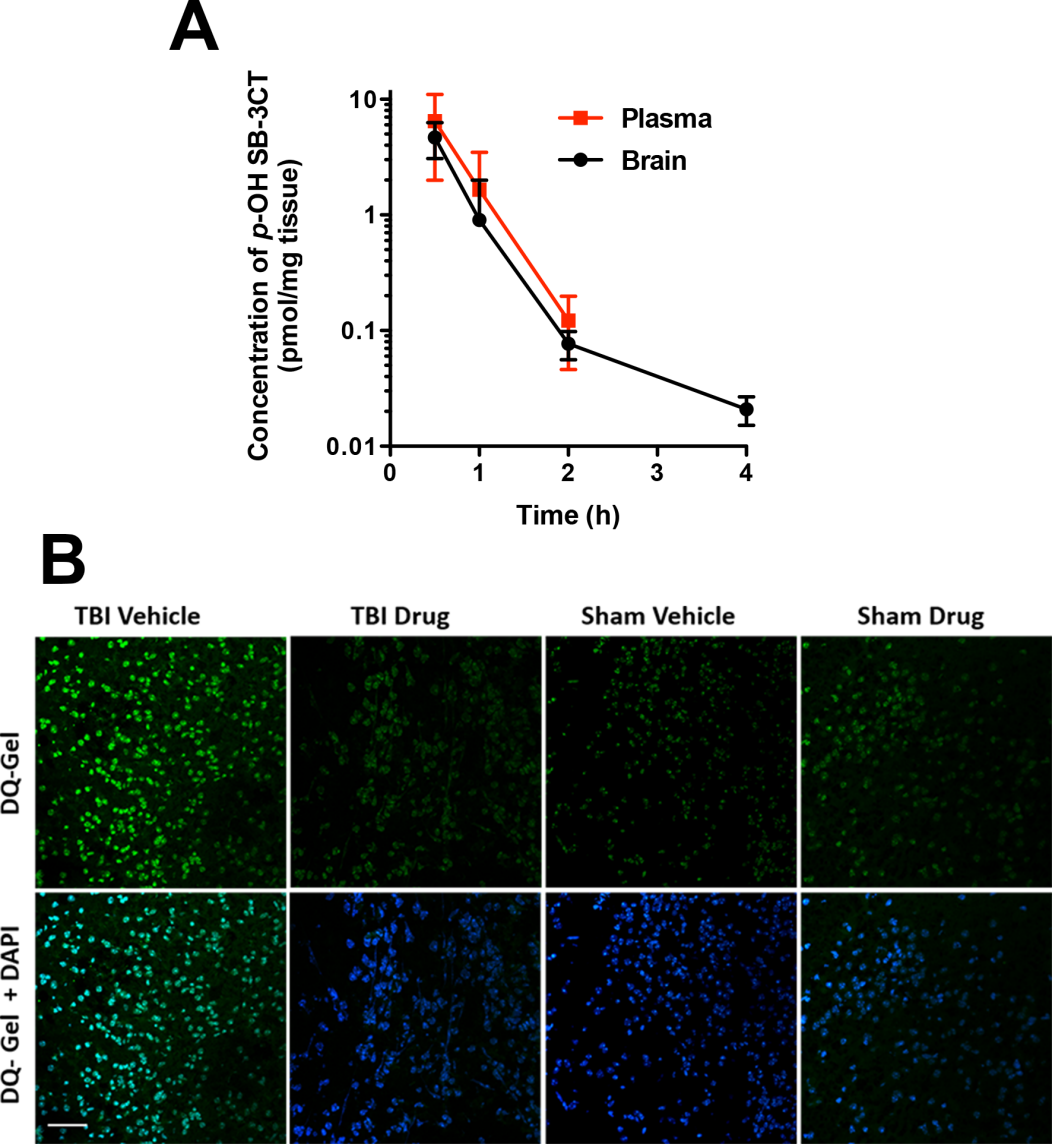
*p*-OH SB-3CT achieved therapeutic concentrations within the immature brain and attenuates gelatinase activity. A) Concentrations are depicted as μM for plasma and pmol/mg tissue for brain (equivalent to μM assuming a density of 1g/mL). Levels of *p*-OH SB-3CT peaked at 30 min after the final dose, in both plasma and brain, and declined by 4 h (n = 3 per time point). Values are mean ± sd. B) Representative images of *in-situ* zymography of brain sections with fluorogenic substrate DQ-Gel (green in top panels) and merged with nuclear staining with DAPI (blue in bottom panels), scale bar is 50 μm.

Based on the negative findings of long-term behavioral and structural outcomes, we considered the possibility that the drug may not cross the blood-brain barrier (BBB). More than 98% of small-molecule therapeutic agents are unable to cross the BBB [68], posing a considerable challenge in the development of therapeutics for the treatment of central nervous system (CNS) diseases. Keeping in mind that the immature brain may differ from adults in terms of barrier properties, absorption rates and other pharmacokinetics [69,70], we evaluated the PK and brain distribution of *p*-OH SB-3CT, specifically after repeated systemic injections in male mice at p21 (25 mg/kg s.c. at 0, 2, and 24 h; n = 3 per group). At 30 min after the final administration, *p*-OH SB-3CT levels peaked in brain tissue at 4.67 ± 1.60 pmol/mg tissue (equivalent to 4.67 μM, assuming a density of 1 g/mL), before declining to 0.0209 ± 0.0058 by 4 h (Table 1). Systemic exposure was 276 μM·min in plasma and 191 pmol·min/mg in brain, for a brain-to-plasma 'area under the curve' (AUC) ratio of 0.693, indicating that *p*-OH SB-3CT crossed the BBB. Levels of *p*-OH SB-3CT in brain samples were above the *K*_i_ values of both MMP-2 and MMP-9 for at least 1 h (Fig 6A). Cumulatively, these results confirm *p*-OH SB-3CT as a quick-acting gelatinase inhibitor that readily crosses the BBB at therapeutically relevant concentrations following systemic administration.

**Table 1:** Concentrations of *p*-OH SB-3CT after multiple-dose s.c. administration.

Time (h)	Brain^*a*^	Plasma^*b*^
0.5	4.67 ± 1.60	6.49 ± 4.49
1	0.905 ± 1.09	1.66 ± 1.82
2	0.0772 ± 0.0211	0.122 ± 0.0762
4	0.0209 ± 0.00578	NQ^*c*^
*AUC*_0-last_ ^*d*^	189	273
*AUC*_0-∞_ ^*d*^	191	276
*Brain*_*AUC*_ */Plasma*_*AUC*_	0.693	

^*a*^ Concentrations in pmol/mg tissue

^*b*^ Concentrations in μM

^*c*^ NQ = not quantifiable

AUC = area under the curve

^*d*^ *AUC* in pmol·min/mg for brain and in μM·min for plasma

To next determine the drug activity, mice received either sham or TBI surgery and were treated with vehicle or *p*-OH SB-3CT (4 mice/group). Brains were dissected from the mice, cryoprotected in mounting medium and sectioned. Sections were treated with DQ- gelatin and imaged. Representative images (Fig 6B) show an increase in gelatinase activity post-injury compared to sham control. Treatment with *p*-OH SB-3CT attenuated gelatinase activity post-injury compared to vehicle control.

### Gelatinase inhibition does not prevent acute cell death in the injured immature brain

Our results indicate that MMP-2 and MMP-9 are upregulated acutely after injury and that *p*-OH SB-3CT crosses the BBB and peaks in the brain at therapeutic concentrations, yet their inhibition does not ameliorate long-term functional or neuroanatomical deficits, suggesting that they may not be key players of long-term outcomes. We therefore evaluated whether they contribute to acute cell death (S3 Fig). A similar degree of cell death was detected at 48 h post-injury in *p*-OH SB-3CT-treated mice compared to vehicle-treated controls, as quantified by staining for TUNEL (Mann-Whitney test, p = 0.2381) and activated caspase-3 (p = 0.6234). These data suggest that early upregulation of MMP-2 and MMP-9 in the acutely injured brain, does not contribute to early secondary pathogenesis.

## Discussion

Using a potent, selective inhibitor of MMP-2 and MMP-9, this study aimed to investigate the role of these gelatinases in chronic pathological and behavioral outcomes after pediatric TBI, with the hypothesis that acute gelatinase activity shapes early progressive neurodegeneration to influence long-term injury consequences. On the contrary, we report for the first time that the gelatinases MMP-2 and MMP-9 are unlikely contributors to progressive neurodegeneration after TBI at p21, based upon our finding that targeted gelatinase inhibition did not ameliorate acute cell death or long-term structural and neurobehavioral and neurocognitive dysfunction. Acute treatment with *p*-OH SB-3CT did not affect hyperactivity, cognitive and social dysfunction at adulthood, following TBI at p21. These data provide compelling evidence that gelatinases, elevated in the acutely injured immature brain, are not key determinants of long-term structural and functional recovery.

### Contrasting role of gelatinases in the p21 versus adult injured brain

The upregulation of both pro- and active forms of MMP-2 and MMP-9 were detected by gelatin zymography at 48 h after injury at p21, consistent with studies in neonatal HI injury [33,35]. The active forms of both gelatinases were detected at lower levels than the pro-enzyme forms, which may be explained by their short half-life in tissue [71,72]. MMP activity is usually tightly regulated by transcriptional induction, pro-enzyme cleavage and binding of tissue inhibitors [46]. Although we did not measure TIMP levels in this study, a dysregulation of TIMPs 1–3 has been reported after adult TBI in both experimental models and patient populations [35,73–75]. In the current study, the robust elevation in both pro- and active forms of MMP-2 and MMP-9 after injury at p21 suggests that the usually tightly-regulated proteolytic environment is unbalanced, likely overwhelming the capacity of these endogenous inhibitors.

Previous experimental models of adult TBI have implicated a central role for elevated MMP activity in the injured brain [21,23–27,30,76], based upon evidence that selective inhibition of the gelatinases affords neuroprotection. Of note, SB-3CT-treatment preserved hippocampal neurons and rescued behavioral and cognitive deficits after TBI by fluid-percussion injury in adult rats [26]. Similarly, in adult models of ischemic stroke, SB-3CT has been found to reduce infarct volumes and ameliorate behavioral and cognitive consequences [23], in part by antagonizing the injury-induced increase in MMP-9 and subsequent laminin degradation [24]. In light of such evidence, translation of the therapeutic targeting of gelatinases into a younger at-risk population requires validation of efficacy in an appropriately-aged model, which prompted the current study in the p21 brain. Further, targeting of MMPs as a potential therapeutic approach is predicated upon the hypothesis that these gelatinases are similarly detrimental in the postnatal developing brain, which may not in fact be the case.

The contrasting findings of our study compared to the prior application of SB-3CT in the adult brain may result from innate properties of the injury response in the developing postnatal brain [44,45]. The immature brain has a reduced antioxidant capacity rendering it potentially more vulnerable to post-traumatic oxidative stress [43], which may result from a plethora of other mediators with diverse effects in addition to the activities of gelatinases. Thus, the inhibition of MMP-2 and MMP-9 in the immature brain may not be sufficient to prevent the cascade of secondary pathogenesis resulting from TBI at this age. Supporting this hypothesis, gelatinase inhibition with SB-3CT administered i.p. at 50 mg/kg at 30 min, 6 h and 12 h after HI injury in rats at p21, similarly failed to render significant neuroprotection, despite demonstrating a reduction in pro-MMP-9 after acute SB-3CT treatment [77]. Strikingly, this study was also conducted in rodents during their third postnatal week of life. Together, these studies provide evidence that gelatinases may have uniquely inconsequential roles in secondary damage after TBI at this age.

### Drug efficacy and pharmacological considerations

*p*-OH SB-3CT was an appropriate inhibitor, as it has a long residence time, longer than TIMP-1 or TIMP-2 complexed with MMP-9 or MMP-2 and avoids untargeted inhibition. The dose of *p*-OH SB-3CT used in the current study was equivalent to the dose of *p*-OH SB-3CT or the parent compound SB-3CT used recently by Hadass and colleagues [25,30], who found a treatment-dependent improvement in functional and neuropathological outcomes after severe TBI in adult mice. *p*-OH SB-3CT is 5-fold and 2.5-fold more potent than the parent compound at inhibiting MMP-2 and MMP-9, respectively, suggesting that this compound treatment may be more effective at targeting the action of gelatinases. We administered *p*-OH SB-3CT by s.c. injection as a more clinically-relevant route, rather than i.p., as previously reported [25], resulting in an initial delay in absorption compared to i.p. administration (30 min versus < 10 min to peak concentrations in the brain). However, the residence times of *p*-OH SB-3CT (how long the drug is bound to the target MMPs) are considerably longer than that of SB-3CT [48,49], resulting in a more prolonged period of inhibition after administration and absorption. Together, and in light of recent efficacy of *p*-OH SB-3CT in adult TBI mice [30], these data suggest that the lack of efficacy of *p-*OH SB-3CT in the current study is not simply a consequence of factors associated with the compound itself, and instead, likely reflects a disconnect between acute gelatinase activity and long-term outcomes in the pediatric brain.

The duration of *p*-OH SB-3CT administration used in this study to target the acute post-injury phase was chosen based upon published literature indicating acute upregulation of active MMPs in the injured adult and neonatal brain [25,35], as well as our own data presented here demonstrating the detection of active and pro-forms of gelatinases at 48 h after TBI at p21. Pharmacokinetic analyses showed that *p-*OH SB-3CT distributes into the brain of healthy immature mice after peripheral administration and therapeutic concentrations required to inhibit the activity of both MMP-2 and MMP-9 were achieved in the young brain. Furthermore, this concentration was sufficient to inhibit the increased gelatinase activity in the injured brain. We also made a conscious decision to administer *p*-OH SB-3CT across a relatively restricted time course of 48 h, in order to minimize potential off-target effects on ongoing brain development at this age [51,52], and avoid potential interference with the beneficial roles of MMPs in wound healing at sub-acute and chronic times post-injury [1,7,78]. Furthermore, *in situ* activation of MMP-9 is tightly regulated and localized, resulting in a short half-life of active MMP-9 [71], hence the treatment was limited to first 48h time-point, where we observe upregulation of active MMP-2 and MMP-9.

### Pleiotropic effects of gelatinases in the developing CNS

Although the detrimental consequences of gelatinases in neuropathology have been well documented, MMPs are ubiquitous proteases with pleiotropic effects, and are necessary for both normal development and wound healing [7,9]. Several MMPs and TIMPs are expressed in the CNS during development, implicating a role in processes of brain maturation including neuronal migration, synaptogenesis, dendritogenesis and myelination [51,52]. Thus, the baseline expression level of MMP-2 and MMP-9 relative to their endogenous inhibitors and activators in the uninjured, immature brain at different ages may influence the response to an insult. After injury, MMPs may promote angiogenesis, neurogenesis and synaptic plasticity. Gelatinase activity is spatially and temporally regulated, and it is likely that MMPs possess different functions across time post-injury, dependent on the cell types that express it, the level of expression, and their location [79,80]. Thus, inhibition of gelatinases may simultaneously curb both detrimental and reparative processes, and treatment strategies targeting MMPs during infancy and childhood should be considered in the context of the many roles that MMPs play during postnatal brain development.

The precise role of MMP-2 in relation to TBI in particular has been poorly defined to date. This gelatinase is thought to contribute to BBB disruption acutely post-injury by degrading junction proteins [81,82], while subsequently promoting axonal regeneration and repair over time [78,83]. Mice with null mutations are useful to discriminate functions of specific MMPs; however, data from these animals should be interpreted with caution, as they often show a compensatory upregulation of other MMPs or related proteins as a result of specific gene deletion [78,84,85]. A recently reported selective MMP-2 inhibitor, that does not inhibit MMP-9, may help resolve the role of MMP-2 in the injured CNS [86].

### Chronic neuropathology and functional deficits after pediatric TBI

We hypothesized that early and brief gelatinase inhibition by *p*-OH SB-3CT administration after early-life TBI would benefit long-term functional and histological recovery, indicating a key role of MMPs in secondary pathogenesis after injury. Surprisingly, and contrary to our hypothesis, both vehicle and drug-treated mice showed similar long-term neurobehavioral and neurocognitive impairments when tested at adulthood (hyperactivity, deficits in task learning, spatial memory and memory retention, and reduced sociability). Of interest, we did see a trend towards a differential effect of the drug on the degree of neurodegeneration, whereby *p*-OH SB-3CT treatment was associated with larger regional volumes in sham mice, but treatment after TBI tended to result in greater bilateral tissue loss in the hippocampus and DG. These findings prompted us to evaluate whether inhibition of gelatinase activity during the acute post-injury phase influences short-term outcomes, as we have recently seen when targeting neutrophil elastase in this model [53]. However, *p*-OH SB-3CT treatment also failed to prevent cell death at 48 h post-injury, a time when MMPs are robustly active, indicating that these gelatinases are not key mediators of acute cell death in this model.

Despite the lack of neuroprotection afforded by *p-*OH SB-3CT, this study serves to further validate previous findings of long-term behavioral and cognitive dysfunction after unilateral TBI to young mice. Consistent with previous studies [41,53], brain-injured mice showed poorer performance in the task learning and memory components of the MWM task, indicating pronounced cognitive deficits at adulthood. Hyperactivity in the open field task was also evident and in line with attention deficit and hyperactivity disorders commonly seen after brain injury in childhood [87,88]. Moreover, behavioral abnormalities were observed by brain-injured mice in the three-chamber task, with a lack of social recognition as previously characterized by our laboratory [42,53,89]. Together, this model consistently produces chronic behavioral and cognitive dysfunction after early life TBI.

Underlying these deficits are neuropathological changes of pronounced neuronal loss and the volumetric reduction of key anatomical regions including the ipsilateral dorsal cortex and hippocampus, at ~2 months post-injury compared to sham-operated controls. Regions contralateral to the impact were also significantly affected at this time, although to a lesser degree compared to ipsilateral structures. These changes are likely attributed to a combination of both mechanical damage from the actual impact, as suggested by a high degree of necrotic and/or apoptotic cell death acutely post-injury, as well as secondary lesion progression [41]. Together with the observed behavioral and cognitive dysfunction, these findings are indicative of a moderate-to-severe injury, evoking the possibility that the lack of efficacy of *p*-OH SB-3CT may be attributed to a high injury severity, such that potentially salvageable tissue was damaged beyond rescue from an early post-injury time point. Future studies examining the role of MMPs across a spectrum of injury severities would address this possibility.

## Conclusion

This is the first study to examine the consequences of acutely upregulated MMP-2 and MMP-9 on long-term behavioral dysfunction after injury to p21 brain. Contrary to our hypothesis, and despite evidence of high inhibitory potency and drug penetration into the young brain, early and brief inhibition of gelatinases by *p-*OH SB-3CT treatment after TBI in the immature mouse did not improve long-term neurobehavioral or neuroanatomical outcomes. In contrast to neuroprotection afforded by targeting MMPs in the adult brain after traumatic or ischemic injuries, our results suggest that MMP-9 and MMP-2 are not key determinants of long-term recovery after injury of the developing brain. Understanding the mechanisms of structural and functional recovery is contingent upon a broader profiling of the acute injury response. Our study highlights that drug therapeutics cannot be designed based on the results in adult TBI models. Furthermore, future studies should be designed to delineate the function of individual MMPs and related proteases in both the healthy and injured immature brain.

## Supporting Information

S1 Fig Experimental design. All mice were treated s.c. with 25 mg/kg *p*-OH SB-3CT or vehicle (65% propylene glycol-35% water) at 2, 4, 24 and 48 h post-injury or sham-operation. Naïve mice were treated with s.c. with 25 mg/kg *p*-OH SB-3CT at 0, 2, and 24h at p21.
(TIF)

S2 Fig Neither injury nor *p*-OH SB-3CT treatment resulted in changes in animal weight or motor function in the MWM. (A) Animals were weighed post-surgery to monitor general health across development, and all mice gained weight over time, with no differences between treatment or injury groups. (B) In the MWM, average swim velocity in the visible platform sessions showed no significant difference between groups (n = 15/group). Analyses by 2-way RM ANOVA. Values represent mean ± sem.
(TIF)

S3 Fig Gelatinase inhibition does not alleviate acute cell death post-injury. Acute cell death was detected by (A) TUNEL staining and (B) cleaved caspase-3 in the brains of vehicle and drug-treated mice at 48 h after TBI at p21. Similar numbers of TUNEL+ and caspase-3+ cells were detected in both groups (n = 6/group). Mann-Whitney tests; individual animals are represented with mean ± sem.
(TIF)
