# Supplementary figures and images for: Early Gelatinase Activity Is Not a Determinant of Long-Term Recovery after Traumatic Brain Injury in the Immature Mouse
Source: PLoS One. 2015 Nov 20;10(11):e0143386. doi: 10.1371/journal.pone.0143386 (PMC4654502; doi:10.1371/journal.pone.0143386)

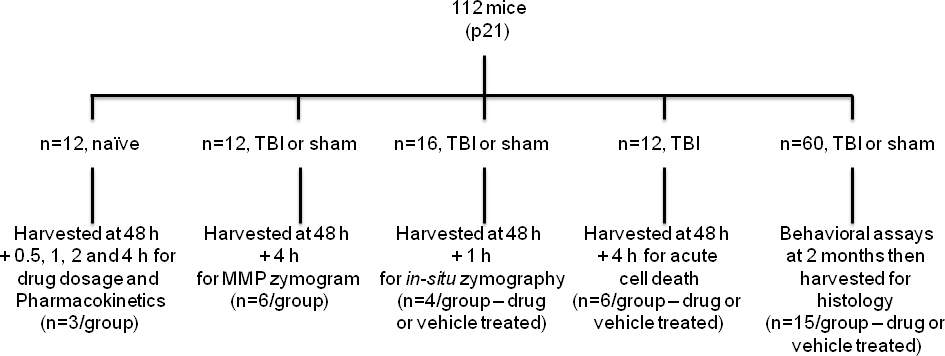

Supplement: S1 Fig — All mice were treated s.c. with 25 mg/kg p-OH SB-3CT or vehicle (65% propylene glycol-35% water) at 2, 4, 24 and 48 h post-injury or sham-operation. Naïve mice were treated with s.c. with 25 mg/kg p-OH SB-3CT at 0, 2, and 24h at p21. (TIF) [file pone.0143386.s001.tif]

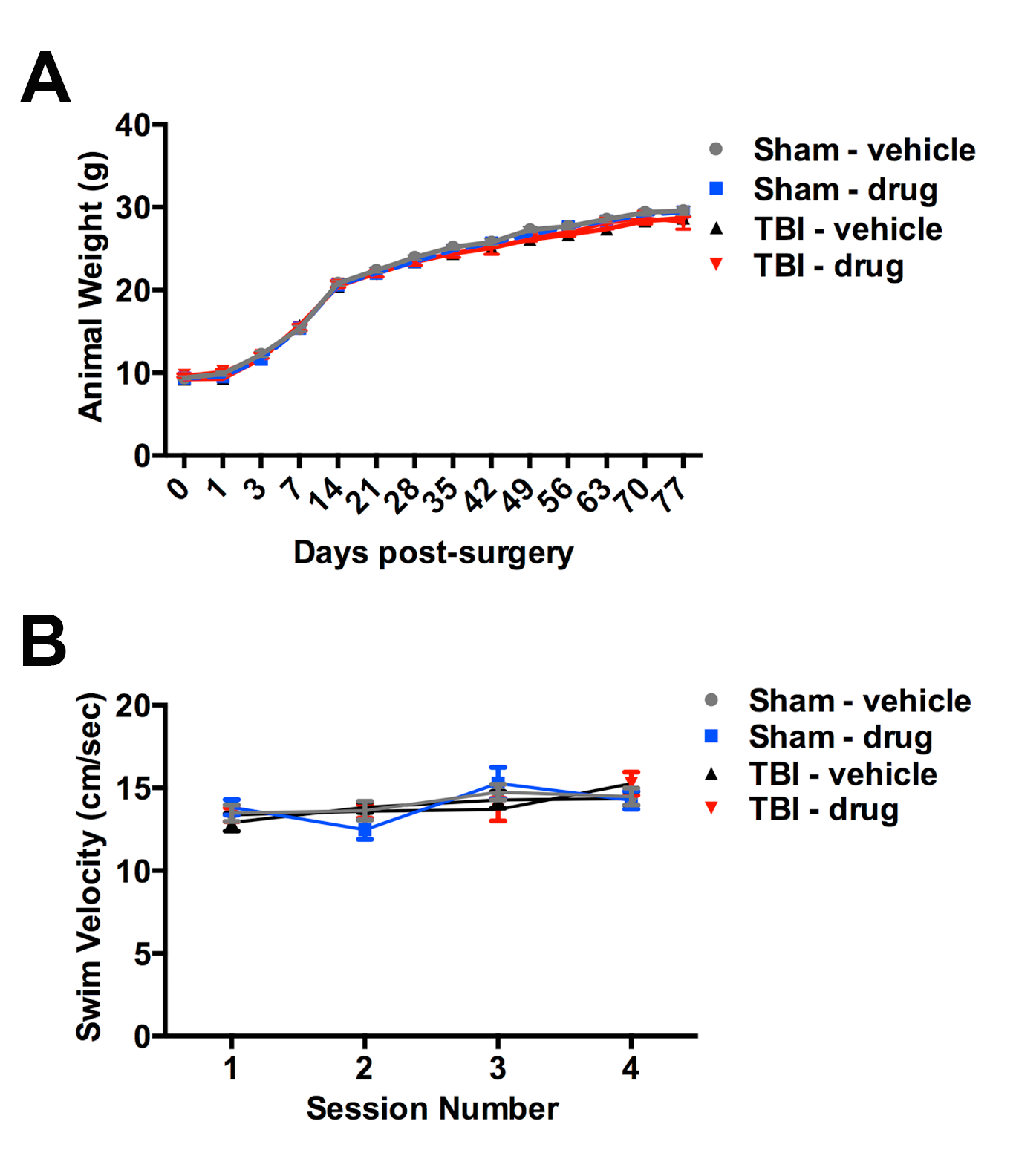

Supplement: S2 Fig — (A) Animals were weighed post-surgery to monitor general health across development, and all mice gained weight over time, with no differences between treatment or injury groups. (B) In the MWM, average swim velocity in the visible platform sessions showed no significant difference between groups (n = 15/group). Analyses by 2-way RM ANOVA. Values represent mean ± sem. (TIF) [file pone.0143386.s002.tif]

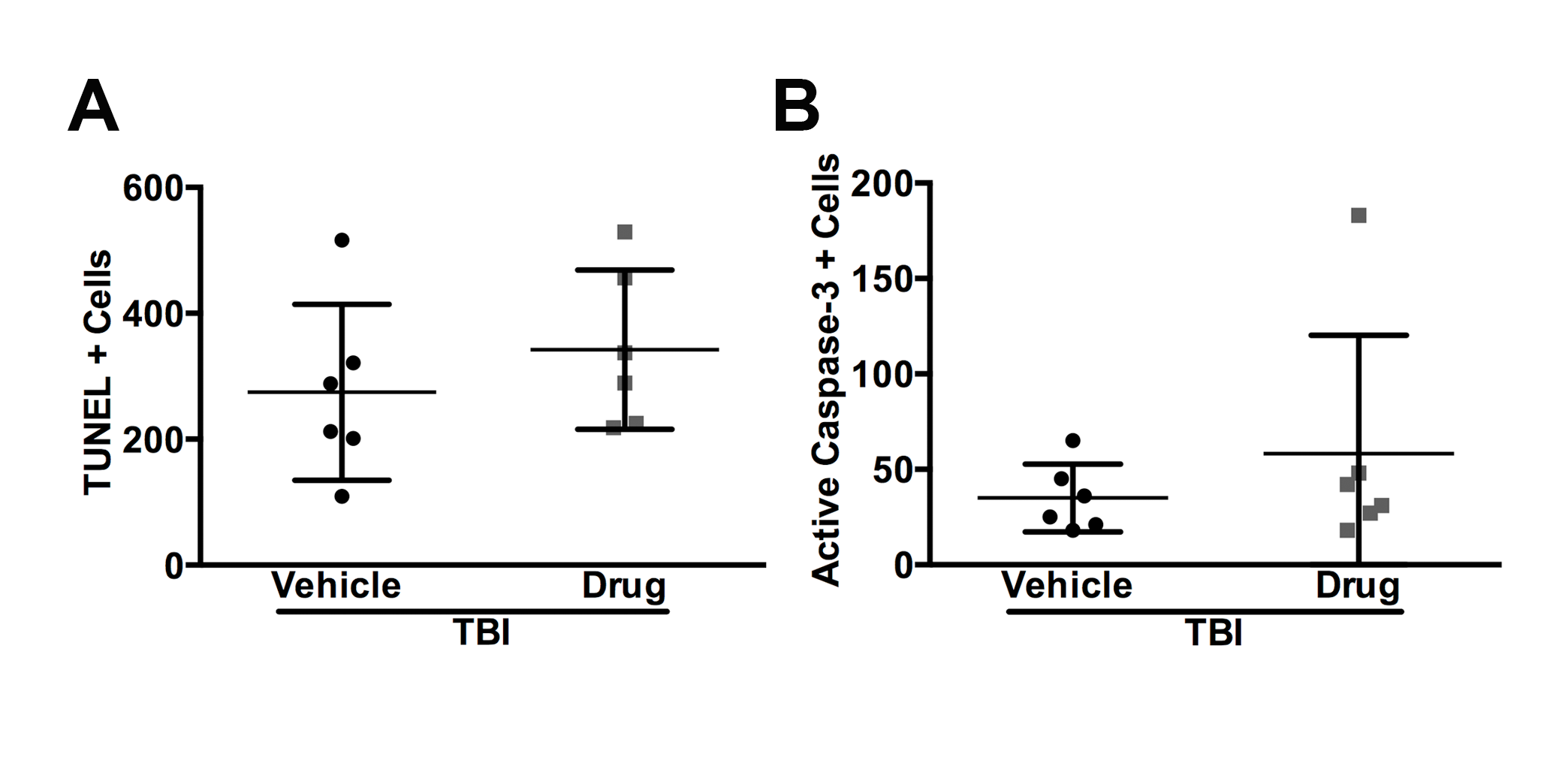

Supplement: S3 Fig — Acute cell death was detected by (A) TUNEL staining and (B) cleaved caspase-3 in the brains of vehicle and drug-treated mice at 48 h after TBI at p21. Similar numbers of TUNEL+ and caspase-3+ cells were detected in both groups (n = 6/group). Mann-Whitney tests; individual animals are represented with mean ± sem. (TIF) [file pone.0143386.s003.tif]
